# Disfunção Ventricular e do Átrio Esquerdo Subclínica em Pacientes com Acromegalia: Um Estudo de Ecocardiograma de Rastreamento de Manchas

**DOI:** 10.36660/abc.20201174

**Published:** 2022-01-11

**Authors:** Hasan Koca, Mevlüt Koc, Hilmi Erdem Sumbul, Yahya Kemal Icen, Erdinc Gulumsek, Fadime Koca, Huseyin Ali Ozturk, Ahmet Oytun Baykan, Onur Kaypakli

**Affiliations:** 1 Adana Health Practice and Research Center Department of Cardiology Adana Turquia Adana Health Practice and Research Center - Department of Cardiology, Adana – Turquia; 2 Adana Numune Training and Research Hospital Adana Turquia Adana Numune Training and Research Hospital, Adana – Turquia; 3 Adana Health Practice and Research Center Department of Internal Medicine Adana Turquia Adana Health Practice and Research Center - Department of Internal Medicine, Adana – Turquia; 4 University of Health Sciences Adana Health Practice and Research Center Adana Turquia University of Health Sciences - Adana Health Practice and Research Center, Adana – Turquia; 5 Ortadogu Private Health Hospital Department of Cardiology Adana Turquia Ortadogu Private Health Hospital - Department of Cardiology, Adana – Turquia; 6 Mustafa Kemal University Tayfur Ata Sokmen Faculty of Medicine Department of Cardiology Hatay Turquia Mustafa Kemal University Tayfur Ata Sokmen Faculty of Medicine - Department of Cardiology, Hatay – Turquia

**Keywords:** Ecocardrgiografia/métodos, Acromegalia, Doenças Crônicas, Doenças Cardiovasculares, Deformação do Miocárdio, Diagnóstico por Imagem, Volume Sistólico

## Abstract

**Fundamento:**

Embora se saiba que a fração de ejeção (FE) do ventrículo esquerdo (VE) medida por eletrocardiograma seja preservada em pacientes com acromegalia, não há informação suficiente sobre deformação longitudinal global e deformação do átrio esquerdo (SLG-VE e SAE).

**Objetivo:**

O objetivo deste estudo foi avaliar as funções do ventrículo esquerdo (VE) e do átrio esquerdo (AE) por ecocardiograma strain (ES) em pacientes com acromegalia.

**Métodos:**

Este estudo incluiu 50 pacientes com acromegalia na forma ativa da doença e 50 controles saudáveis com idade, sexo e área de superfície corporal similares. Além dos ecocardiogramas de rotina, medições de SLG-VE e SAE foram realizadas com o ES.

**Resultados:**

Os valores dos SAE e SLG-VE foram significativamente mais baixos em pacientes com acromegalia (p<0,05 para todos). Na análise bivariada, a pressão arterial sistólica, o pró-hormônio N-terminal do peptídeo natriurético cerebral, o fator de crescimento semelhante à insulina tipo 1, e detectou-se que os níveis de IMVE tinham correlação positiva com SAE e SLG-VE (p<0,05). O nível de IGF-1 tinha forte correlação com SAE e SLG-VE (p<0,001 e β=0,5 vs. p<0,001 e β=0,626, respectivamente); 48% dos pacientes com acromegalia têm SLG-VE reduzido (<20%). O índice de massa do ventrículo esquerdo (IMVE) determina independentemente a presença de SLG-VE reduzido, e cada 1g/m^2^ de aumento no nível de IMVE aumenta a probabilidade de redução de SLG-VE em 6%.

**Conclusão:**

Embora a fração de ejeção de VE seja normal em pacientes com acromegalia, os valores de SAE e SLG-VE são significativamente mais baixos. Além do aumento em IMVE, outro achado do envolvimento cardíaco pode ser a redução de SAE e SLG-VE. Portanto, além do ecocardiograma de rotina, SAE e SLG-VE podem ser úteis para avaliar os sinais iniciais de envolvimento cardíaco antes da ocorrência de alterações cardíacas irreversíveis.

## Introdução

A acromegalia é uma doença crônica caracterizada pelo aumento da síntese do fator de crescimento semelhante à insulina tipo 1 (IGF-1) no fígado devido a um adenoma pituitário secretor de hormônio de crescimento (GH), e síntese excessiva de proteína e crescimento tecidual excessivo devido a esses hormônios.^1^ Níveis altos de IGF-1 crônicos causam alterações estruturais e funcionais específicas.^1^ Se não forem tratados, levam à morte, cuja causa mais comum são as doenças cardiovasculares (CV).^1 , 2^

Strain é uma medida de deformação em relação a uma potência em uma substância. Ele é definido em dimensões radiais, circunferenciais e longitudinais. A avaliação da deformação miocárdica por ecocardiograma strain (ES), em termos de um ecocardiograma de rastreamento de manchas bidimensional (2D-STE) ou imagem por Doppler tecidual (strain convencional) pode oferecer informações incomparáveis sobre as funções ventriculares regionais e globais.^3^ Imagens de deformação podem detectar até as mínimas alterações funcionais e garantir um diagnóstico em fase inicial. Os parâmetros de deformação do átrio esquerdo (AE) e deformação longitudinal global do ventrículo esquerdo (SLG-VE) demonstraram estar fortemente correlacionados às funções sistólicas do AE e do VE em cenários clínicos diferentes, respectivamente.^4 - 8^ Demonstrou-se que muitos pacientes com fração de ejeção de VE (FEVE) normal têm redução da função sistólica do VE com o uso do SLG-VE.^3 , 9 , 10^

Em pacientes com acromegalia, a função sistólica é avaliada com FEVE. Entretanto, deficiências em FEVE só são vistas em etapas posteriores da doença e na minoria dos pacientes.^11 - 14^ Recentemente, um número limitado de estudos avaliou a deformação de AE e SLG-VE em pacientes com acromegalia e FEVE preservada.^5 - 8^ Foram obtidos resultados contraditórios nesses estudos. Detectou-se, em dois estudos conduzidos pelos mesmos autores, a redução do SLG-VE;^8 , 9^ enquanto outro estudo relatou que o SLG-VE era semelhante ao dos pacientes de controle saudáveis.^5^ A avaliação das medidas de deformação do AE em pacientes com acromegalia foi realizada com 3D-STE em um estudo; entretanto, não se obtiveram informações claras sobre a alteração da deformação global no AE.^8^

Devido à viabilidade de medições simultâneas da deformação de AE e SLG-VE, além do ecocardiograma tradicional, este estudo teve o objetivo de avaliar as funções do VE e do AE com ES em pacientes com acromegalia ativa e FEVE preservada.

## Métodos

### População do estudo

**Figura 1:**
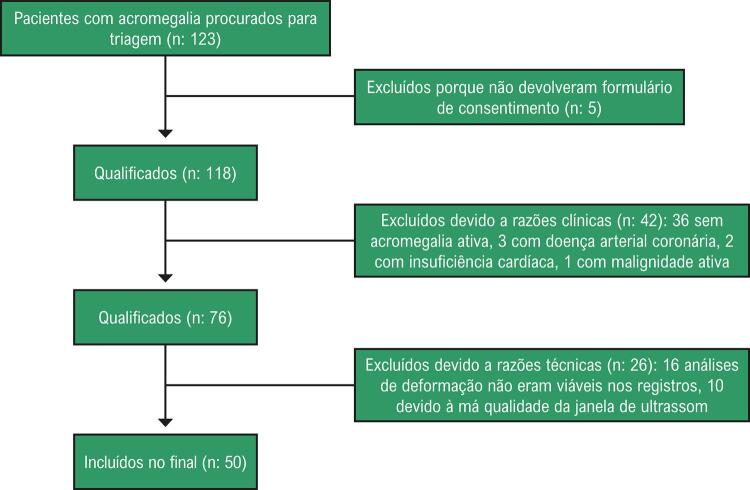
– Fluxograma para inclusão e avaliação de pacientes no estudo.

Neste estudo transversal, 50 pacientes (33 do sexo masculino, 17 do sexo feminino; média de idade de 46,1 ± 6,2 anos) com acromegalia ativa (1-Pacientes recorrentes, 2-Pacientes pós-cirurgia sem remissão, 3-Pacientes em tratamento médico sem remissão) e idade, sexo, índice de massa corporal (IMC) e área de superfície corporal (ASC) correspondentes a 50 pacientes de controle saudáveis (31 do sexo masculino, 19 do sexo feminino; média de idade: 44,6 ± 5,1 anos). Pacientes acima dos 18 anos de idade com acromegalia ativa foram cadastrados no estudo. Os pacientes incluídos no estudo são apresentados no fluxograma ( Figura 1 ). Informações de diretrizes atuais foram utilizadas para o diagnóstico, o tratamento e a classificação de pacientes com acromegalia.^1^ A remissão da acromegalia foi definida como um GH sérico suprimido por glicose abaixo de 0,38 μg/litro (<1 mU/litro), um GH sérico abaixo de 1,9 μg/litro(<5 mU/litro), e IGF-1 normal para a idade.^1^ Pacientes com histórico de doença arterial coronariana (DAC) e infarto do miocárdio, arritmia cardíaca, insuficiência cardíaca sistólica ou FEVE <50%, doença de válvula cardíaca, embolia pulmonar, disfunção da tireoide, gravidez (comprovada ou suspeita), malignidade, e disfunção renal e hepática, e pacientes que se recusaram a participar do estudo não foram incluídos. O comitê de ética local aprovou o protocolo do estudo (Comitê de Ética da Faculdade de Medicina da Universidade Çukurova, 03.05.2019-88), e foi obtido o consentimento informado por escrito de cada um dos participantes.

Após a avaliação do histórico médico detalhado e um exame físico completo, as características de linha de base dos pacientes, incluindo idade, sexo, hipertensão (HT), diabetes mellitus (DM), hiperlipidemia, tabagismo atual, histórico familiar de doenças cardíacas e medicamentos, foram registradas para todos os pacientes. Os parâmetros de IMC e ASC dos participantes foram calculados.

Os níveis de glicose, nitrogênio ureico sanguíneo, creatinina, colesterol total, colesterol de lipoproteína de baixa densidade, colesterol de lipoproteína de alta densidade, triglicérides, aspartato aminotransferase, alanina aminotransferase, leucócitos, hemoglobina, proteína C-reativa sensível alta, e peptídeo natruriético pró-cérebro N-terminal (NT-proBNP) foram medidos utilizando-se um analisador químico automático (Abbott Aeroset, MN, EUA) com os kits comerciais apropriados (Abbott). O GH sérico foi avaliado por um analisador químico automático (Abbott Aeroset, MN, EUA) utilizando-se os kits comerciais apropriados (Abbott) e o valor de referência do GH estava entre 0,014 -5,219 ng/ml. O IGF-1 sérico total foi avaliado por um analisador químico automático (Abbott Aeroset, MN, EUA) utilizando-se os kits comerciais apropriados (Abbott) e o valor de referência do IGF-1 varia de acordo com idade e sexo. Os níveis de GH e IGF-1 foram medidos ao mesmo tempo do exame ecocardiográfico para cada um dos sujeitos.

### Avaliação ecocardiográfica

A avaliação ecocardiográfica foi feita utilizando-se um transdutor de 2,5-3,5 MHz EPIQ 7C (Philips Healthcare 3000 Minuteman Road, Andover, MA, EUA). A avaliação ecocardiográfica foi realizada durante a primeira semana para pacientes que atenderam aos critérios de inclusão. Avaliações ecocardiográficas de todos os pacientes foram realizadas em posição de decúbito lateral esquerdo com monitoramento de pressão arterial e eletrocardiográfico. Todas as imagens foram obtidas em pelo menos 3 ciclos repetitivos do eixo paraesternal longo e curto padrão, cortes apicais de 4 câmaras, de 5 câmaras, e 2 câmaras de acordo com as sugestões da *American Society of Echocardiography* (Sociedade Americana de Ecocardiografia).^15^ O diâmetro diastólico do VE, o diâmetro sistólico do VE, a espessura do septo interventricular (SIV), a espessura da parede posterior (PP), e o diâmetro diastólico do AE foram medidos a partir da imagem do eixo longo paraesternal de janelas de imagem bidimensionais. A fórmula de Devereux foi utilizada para a medição da massa do VE.^16^ Em seguida, o índice de massa do VE (IMVE) foi calculado dividindo-se a massa do VE pela ASC. O valor do IMVE >115 gr/m^2^em homens e >95 gr/m^2^ em mulheres foram considerados hipertrofia do VE.^17^

No procedimento de ES, todos os pacientes tinham ritmo sinusal normal. Os parâmetros de deformação miocárdica de AE e VE foram calculados utilizando-se STE sobre imagens em escala de cinza bidimensionais. Foram registradas imagens apicais de 4 câmaras (A4C), apicais de 2 câmaras (A2C) e apicais de 3 câmaras (A3C) em escala de cinza bidimensionais após a expiração após prender o fôlego. Pelo menos três ciclos cardíacos foram registrados para cada imagem, e prestou-se atenção para se considerar pelo menos 60-80 fps, de acordo com as diretrizes da *European Society of Cardiology* (Sociedade Europeia de Cardiologia).^18^ Segmentos com qualidade de imagem insuficiente e ciclos cardíacos contendo batimentos prematuros foram excluídos das medições.

**Figura 2:**
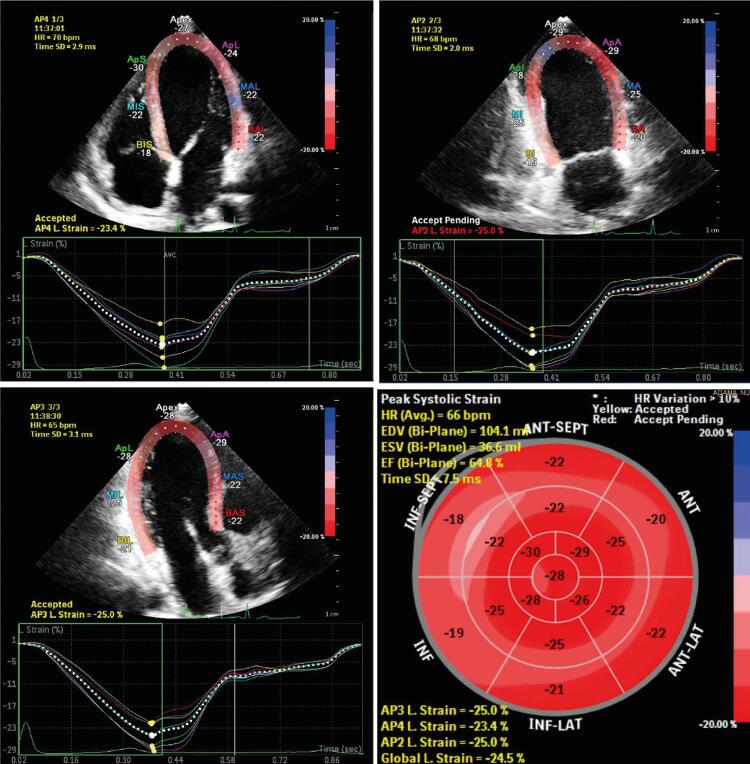
– Medição da deformação longitudinal global do ventrículo esquerdo (SLG-VE) por ecocardiograma strain em pacientes com acromegalia.

O software QLAB versão 10.5 (Philips, Andover, MA, EUA) foi utilizado para as análises de VE e AE. O software seguiu automaticamente os movimentos da parede durante todo o ciclo cardíaco após o endocárdio do VE ter sido marcado quadro a quadro pelo método de desenho manual (rastreamento manual) nas imagens bidimensionais. Os valores de SLG-VE foram calculados a partir das imagens com 2D-STE. Após a marcação manual de 2 partes basais e 1 parte apical do VE, as bordas endocárdicas restantes foram automaticamente marcadas pelo software e a borda epicárdica apropriada também foi automaticamente desenhada. Quando os contornos de VE desenhados automaticamente não eram adequados para análise, as bordas foram corrigidas manualmente para permitir uma análise adequada. Após a análise, o software dividiu os registros de A2C, A3C, A4C do VE em modelos de seis segmentos, e o modelo de 18 segmentos foi usado para calcular o SLG-VE ( Figura 2 ).

**Figura 3:**
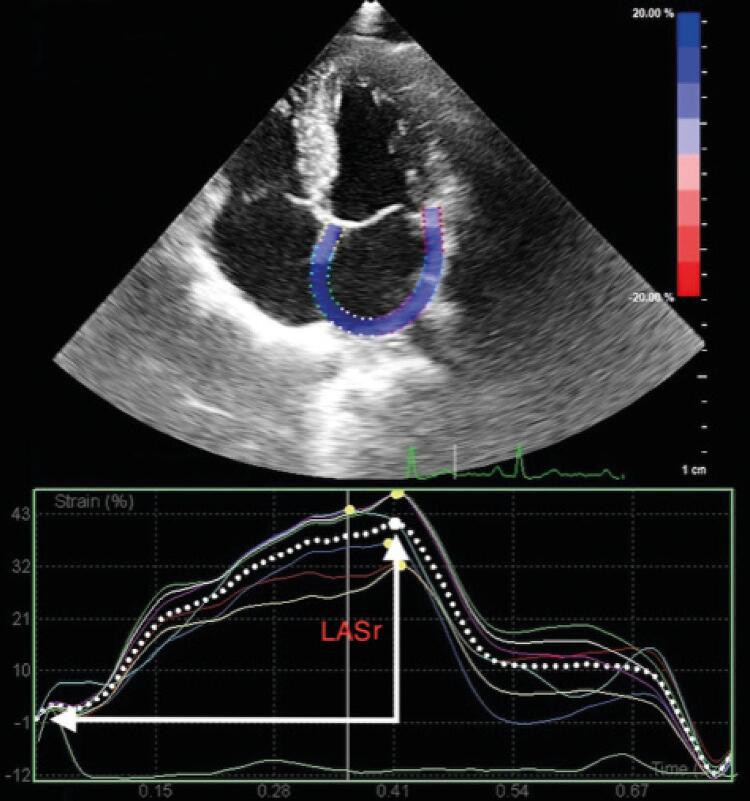
– Medição da deformação longitudinal global do ventrículo esquerdo (SLG-VE) por ecocardiograma strain em pacientes com acromegalia.

Parâmetros de deformação miocárdica do AE também foram calculados a partir das imagens 2D-STE utilizando-se o software da deformação do VE.^19^ Foram obtidas imagens do AE em corte de quatro câmeras utilizando-se marcos anatômicos-padrão para garantir a aquisição ideal e evitar o encurtamento com ecocardiograma bidimensional convencional, em índices de quadros relativamente altos (60–80 fps). O contorno endocárdico do AE foi iniciado na borda endocárdica do anel mitral até a borda endocárdica do AE, extrapolando pelas veias pulmonares, e/ou orifícios de apêndices de AE, até o anel mitral oposto por um radiologista experiente, cego em relação às informações clínicas. O software então gerou automaticamente uma silhueta de AE epicárdica, que delineou uma região de interesse em cada um dos cortes apicais. O ajuste manual da região de interesse foi permitido para incluir toda a camada miocárdica do AE, seguido de um rastreamento segmentar automático. Após o rastreamento, os índices de deformação do AE, tais como da deformação longitudinal e suas primeiras curvas SR de derivação foram obtidas a partir de uma vista apical de quatro câmaras.^20^ Utilizamos uma onda R como ponto de partida (ligação R-R) para análise da deformação. A deformação longitudinal e as curvas de índice de deformação foram geradas em todos os segmentos, e a média dos segmentos foi calculada para os pontos de tempo correspondentes ( Figura 3 ). Utilizando essas curvas, a deformação do AE (SAE) e os índices de deformação de pico sistólico (RSAE) foram calculados. O SAE e o RSAE representam a função de reservatório do AE. Todas as imagens ecocardiográficas foram armazenadas digitalmente e analisadas off-line, com as medidas de deformação realizadas por um cardiologista experiente, cego em relação aos dados, utilizando análise de software Philips QLAB versão 10.5.

### Análises estatísticas

As análises estatísticas foram realizadas utilizando-se o software SPSS, versão 23.0 (SPSS Inc., Chicago, Illinois, EUA). Os dados foram expressos como média ± DP para variáveis contínuas e porcentagens para variáveis categóricas. O teste de Shapiro-Wilk foi utilizado para testar a normalidade e um p-valor >0,05 foi definido como dado normalmente distribuído. Variáveis contínuas que apresentaram distribuição normal foram comparadas utilizando-se o teste T de Student e ANOVA, enquanto o teste U de Mann-Whitney e o teste de Kruskal-Wallis foram usados para amostras não distribuídas normalmente. Variáveis categóricas e frequências foram comparadas pelo teste Qui-quadrado. A significância estatística foi definida como um p-valor <0,05 para todas as comparações. Neste estudo, os parâmetros que foram diferentes em pacientes com <20% para SLG-VE foram encontrados em análise univariada. Portanto, retroativamente: A análise de regressão logística (RL) foi realizada para se determinar os parâmetros que determinaram de forma independente pacientes com SLG-VE <20%. As correlações de Pearson e Spearman foram usadas para examinar a relação entre variáveis contínuas. Variáveis com um p-valor <0,05 na análise bivariada foram testadas na análise de regressão linear. Os resultados foram expressos como p-valor e razão de risco (RR) no IC de 95%.

## Resultados

Foram incluídos no estudo cinquenta e oito pacientes com acromegalia ativa. Foram excluídos do estudo oito pacientes que atenderam aos critérios de exclusão e que não puderam realizar o exame ecocardiográfico ideal. Dos pacientes incluídos no estudo, 45 eram pacientes recorrentes de acromegalia. Dos demais pacientes, três eram pacientes pós-cirurgia sem remissão, e dois estavam fazendo tratamento médico sem remissão. Os dados do estudo foram divididos em dois grupos, com e sem acromegalia (pacientes de controle saudáveis). Os coeficientes kappa de Cohen que avaliaram a variabilidade interobservador e intraobservador estavam acima de 90% de todas as medições de ecocardiogramas.

### Dados demográficos, clínicos e laboratoriais

Quando os dados demográficos foram comparados entre os grupos do estudo: idade, sexo, IMC e ASC eram semelhantes entre os grupos. Determinou-se que a frequência de HT e DM em pacientes com acromegalia era de 28% e 32%, respectivamente. Em termos de parâmetros clínicos, as pressões arteriais sistólica e diastólica, e a frequência cardíaca eram mais altas em pacientes com acromegalia. Identificou-se que os níveis de glicemia plasmática, NT-proBNP, IGF-1 e hormônio de crescimento eram significativamente mais altos em pacientes com acromegalia. Parâmetros de outros laboratórios eram semelhantes entre os dois grupos ( Tabela 1 ).

**Tabela 1:** – Parâmetros demográficos e laboratoriais de pacientes com acromegalia em pacientes de controle saudáveis

Variável	Pacientes com acromegalia n=50	Controles saudáveis n=50	p
Idade (anos)	46,1 ± 6,2	44,6 ± 5,1	0,295
Sexo (feminino)	17	19	0,418
Hipertensão, n (%)	14 (28%)	–	–
Diabetes mellitus, n (%)	16 (32%)	–	–
Fumante, n (%)	15 (30%)	–	–
Hiperlipidemia, n (%)	7 (14%)	–	–
Pressão arterial sistólica (mmHg)	130 ± 19	110 ± 10	<0,001
Pressão arterial diastólica (mmHg)	81 ± 11	67 ± 6,4	<0,001
Frequência cardíaca (pulsos/minuto)	81 ± 11	67 ± 4,1	<0,001
Índice de massa corporal (kg/m^2^)	28,1 ± 2,3	27,6 ± 1,6	0,164
Área da superfície corporal (m^2^)	2,01 ± 0,10	2,00 ± 0,09	0,569
Leucócito (µL)	7,3 ± 1,9	7,5 ± 1,6	0,656
Hemoglobina (gr/dL)	13,1 ± 1,8	12,9 ± 1,2	0,420
Plasma glicemia (mg/dL)	109 ± 23	92 ± 5,6	<0,001
Nitrogênio ureico sanguíneo (mg/dL)	32,9 ± 16,6	29,5 ± 4,1	0,149
Creatinina (mg/dL)	0,75 ± 0,42	0,64 ± 0,10	0,138
Colesterol total (mg/dL)	197 ± 59	217 ± 60	0,095
Lipoproteína de baixa densidade (mg/dL)	135 ± 45	148 ± 44	0,157
Lipoproteína de alta densidade (mg/dL)	44,3 ± 15,3	48,2 ± 8,1	0,125
Triglicérides (mg/dL)	165 ± 77	191 ± 108	0,180
Aspartato aminotransferase (u/L)	20,6 ± 7,4	18,9 ± 3,4	0,143
NT-proBNP (pg/mL)	365 ± 297	74 ± 6,7	<0,001
PCR-as (mg/dL)	1,69 ± 1,35	0,43 ± 0,31	<0,001
Alanina aminotransferase (u/L)	16,8 ± 8,9	15,9 ± 2,9	0,298
IGF-1 (ng/dL)	376 ± 181	72 ± 7,5	<0,001
Hormônio do crescimento (ng/mL)	9,21 ± 14,4	1,01 ± 0,52	<0,001

*PCR-as: Proteína C reativa alta sensível; IGF-1: Fator de crescimento semelhante à insulina tipo 1; NT-proBNP: Peptídeo natriurético pró-cérebro N-terminal.*

### Dados ecocardiográficos

**Figura 4:**
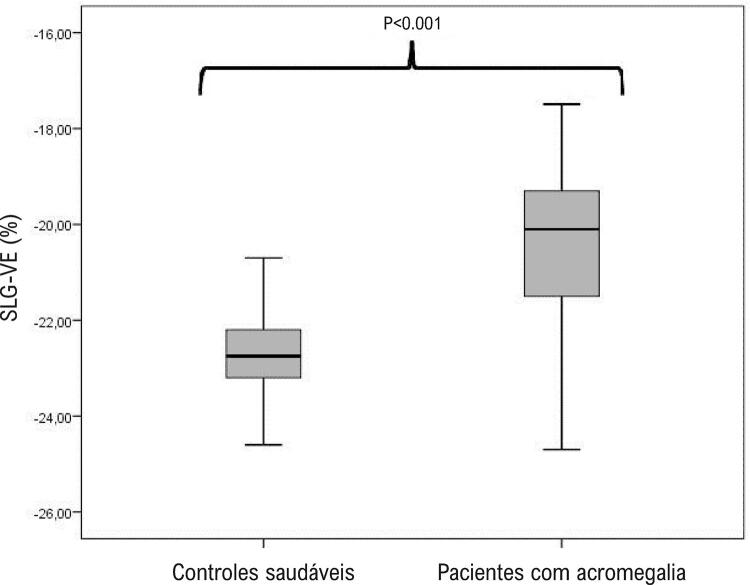
– O gráfico de caixa demonstrou a deformação longitudinal global do ventrículo esquerdo (SLG-VE) em pacientes com acromegalia e em pacientes de controle saudáveis.

**Figura 5:**
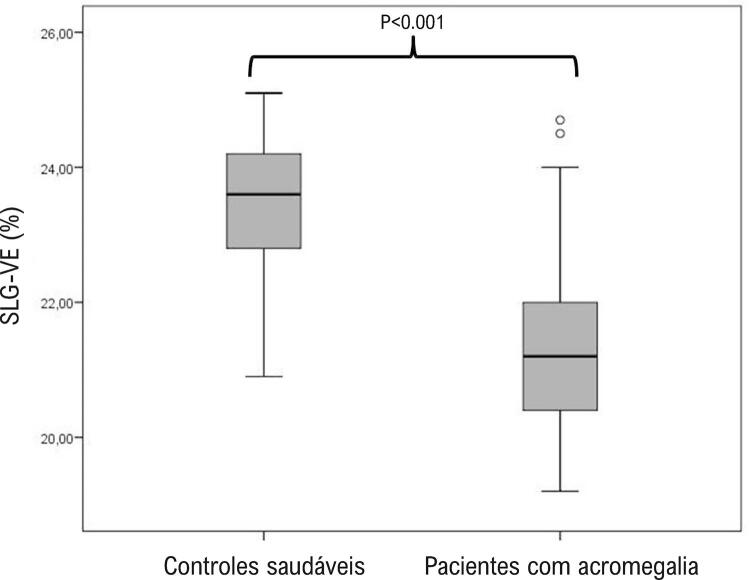
– O gráfico de caixa demonstrou o pico positivo de deformação de AE (SAE) em pacientes com acromegalia e em pacientes de controle saudáveis.

Identificou-se que os valores de SIV, espessura diastólica final da PP, e IMVE eram significativamente mais altos em pacientes com acromegalia ( Tabela 2 ). Determinou-se que os diâmetros de VE e os valores de FEVE eram semelhantes entre os grupos com e sem acromegalia. Os valores de SAE e SLG-VE são significativamente mais baixos em pacientes com acromegalia ( Figura 4 - 5 ).

**Tabela 2:** – Parâmetros ecocardiográficos de pacientes com acromegalia em pacientes de controle saudáveis

Variável	Pacientes com acromegalia n=50	Controles saudáveis n=50	p
Espessura diastólica final de SIV (mm)	12,3 ± 1,92	9,9 ± 1,21	<0,001
Espessura diastólica final de PP (mm)	11,9 ± 1,32	9,7 ± 1,01	<0,001
Dimensão diastólica final do VE (mm)	46,7 ± 4,5	47,3 ± 4,3	0,516
Dimensão sistólica final do VE (mm)	31,1 ± 4,2	31,5 ± 4,2	0,656
Dimensão diastólica final do AE (mm)	35,3 ± 4,2	33,1 ± 2,6	0,002
Fração de ejeção VE (%)	57,8 ± 4,1	58,9 ± 5,3	0,259
Índice de massa do VE (gr/m^2^)	108 ± 28	82± 17	<0,001
Hipertrofia do VE, n (%)	25 (50%)	0 (0%)	<0,001
SAE (%)	21,5 ± 1,36	23,5 ± 1,06	<0,001
SLG-VE (%)	– 20,4 ± 1,45	– 22,8 ± 0,83	<0,001
SLG-VE <20, n (%)	24 (48%)	0 (0%)	<0,001

*SIV: Septo interventricular; AE: Átrio esquerdo; SLG-VE: Deformação longitudinal global do ventrículo esquerdo; VE: ventrículo esquerdo; PP: Parede posterior; SAE: deformação de AE no pico positivo.*

Quando o valor limite foi tomado como <20% para SLG-VE, detectou-se que os níveis de SLG-VE eram reduzidos em 48% do grupo com acromegalia. Quando os dados demográficos, clínicos e laboratoriais de pacientes com acromegalia SLG-VE normal e reduzida foram comparados, determinou-se que pacientes com SLG-VE baixo tinham índices mais altos de HT (45,8% x 11,5% e p = 0,008) e valores mais altos de IMVE (123 gr/m^2^ x 94,6 gr/m^2^ e p <0,008). Na análise de regressão logística multivariada, detectou-se que o valor de IMVE e os níveis de IGF-1 fazem a previsão independente do valor de SLG-VE reduzido (p = 0,003, RC: 1,060 e IC: 1,019 - 1,102 e p = 0,012, RC: 1,056 e IC: 1,023 - 1,098). De acordo com essa análise, cada aumento de 1 gr/m^2^ no valor de IMVE e cada aumento de 1 ng/dL nos níveis de IGF-1 aumenta a probabilidade de diminuição de SLG-VE em 6% e 5,6%, respectivamente.

### Determinação da medição de deformação do AE

**Figura 6:**
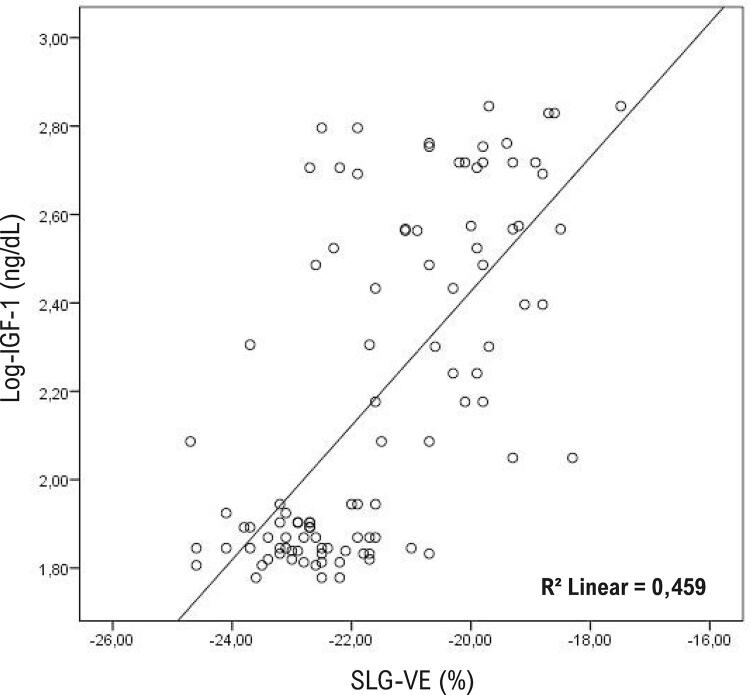
– Gráfico de dispersão da relação do pico positivo de deformação de AE (SAE) com fator de crescimento semelhante à insulina tipo 1 (IGF-1). Foi obtida uma escala logarítmica na base 10 de IGF-1.

A análise de correlação foi realizada para determinar os parâmetros associados à deformação do AE. Os parâmetros relacionados a SAE na análise de correlação foram resumidos na Tabela 3 . A análise de regressão linear foi realizada para determinar a presença de relações independentes de SAE. Na análise de regressão linear, detectou-se uma associação positiva e significativa entre PA sistólica, NT-proBNP, IGF-1, diâmetro diastólico final do AE e IMVE, e SAE. Estatisticamente, a correlação mais forte identificada foi entre SAE e os níveis de IGF-1 ( Tabela 3 e Figura 6 ).

**Tabela 3:** – Os parâmetros associados ao SLG-AE e à análise por regressão linear para parâmetros significativamente correlacionados a SAE

	Análise univariada		Análise multivariada	
	p	r	p	β
Pressão arterial sistólica	<0,001	0,427	0,001	0,278
Pressão arterial diastólica	<0,001	0,362	0,470	0,102
Frequência cardíaca	<0,001	0,360	0,840	0,023
Glicemia plasmática	<0,001	0,418	0,255	0,133
Creatinina	0,018	0,225	0,712	0,064
NT-proBNP	<0,001	0,445	0,013	0,237
IGF-1 (ng/dL)	<0,001	0,531	<0,001	0,531
Hormônio do crescimento (ng/mL)	0,025	0,225	0,408	0,096
Dimensão diastólica final do AE	<0,001	0,662	<0,001	0,378
Índice de massa do VE	<0,001	0,623	<0,001	0,503

*AE: Átrio esquerdo; SAE: deformação de AE no pico positivo; VE: Ventrículo esquerdo; IGF-1: Fator de crescimento semelhante à insulina tipo 1; NT-proBNP: Peptídeo natriurético pró-cérebro N-terminal. R^2^_padronizado_=0,684 e p<0.001 análises multivariadas.*

### Determinação de parâmetros relacionados a SLG-VE

**Figura 7:**
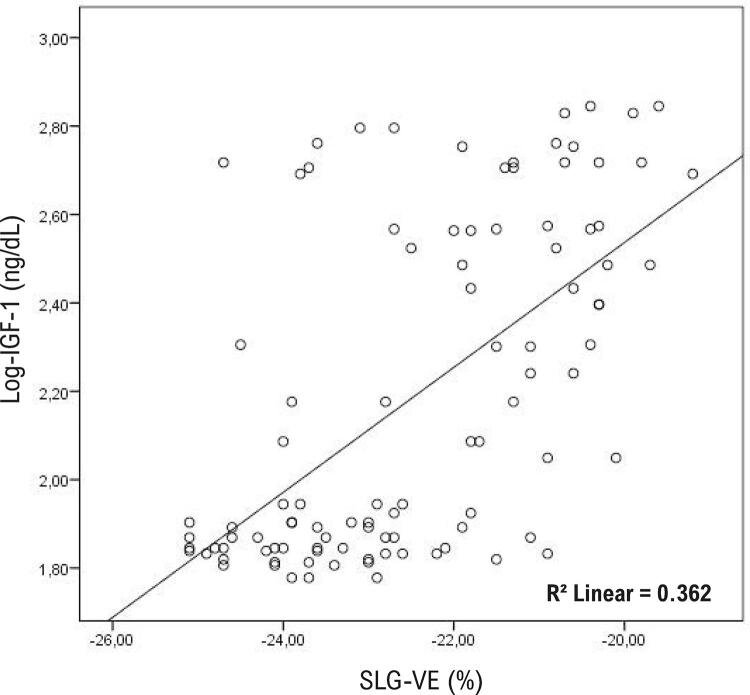
– Gráfico de dispersão da relação da deformação longitudinal global do ventrículo esquerdo (SLG-VE) com fator de crescimento semelhante à insulina tipo 1 (IGF-1). Foi obtida uma escala logarítmica na base 10 de IGF-1.

A análise de correlação foi realizada para determinar os parâmetros associados à deformação do SLG-VE. Os parâmetros relacionados a SLG-VE na análise de correlação foram resumidos na Tabela 4 . A análise de regressão linear foi realizada para determinar a presença de relações independentes de parâmetros relacionados a SLG-VE. Na análise de regressão linear, detectou-se uma associação positiva e significativa entre pressão arterial sistólica, NT-proBNP, IGF-1, diâmetro diastólico final do AE e IMVE, e valor de SLG-VE. Estatisticamente, a correlação mais forte identificada foi entre SLG-VE e os níveis de IGF-1 ( Tabela 4 e Figura 7 ).

**Tabela 4:** – Os parâmetros associados ao SLG-VE e à análise por regressão linear para parâmetros significativamente correlacionados a SLG-VE

	Análise univariada		Análise multivariada	
	p	r	p	β
Idade	0,026	0,223	0,844	0,017
Índice de massa corporal	0,033	0,213	0,256	0,092
Pressão arterial sistólica	<0,001	0,509	<0,001	0,300
Pressão arterial diastólica	<0,001	0,462	0,605	0,076
Frequência cardíaca	<0,001	0,408	0,426	0,081
Glicemia plasmática	<0,001	0,442	0,172	0,146
Creatinina	0,015	0,243	0,263	0,090
NT-proBNP	<0,001	0,478	0,011	0,176
IGF-1	<0,001	0,626	<0,001	0,626
Hormônio do crescimento (ng/mL)	<0,001	0,429	0,050	0,207
Dimensão diastólica final do AE	0,001	0,341	0,009	0,199
Índice de massa do VE	<0,001	0,623	<0,001	0,548

*IGF-1: Fator de crescimento semelhante à insulina tipo 1; NT-proBNP: Peptídeo natriurético pró-cérebro N-terminal; SLG-VE: Deformação longitudinal global do ventrículo esquerdo. R^2^_padronizado_=0,641 e p<0.001 análises multivariadas.*

## Discussão

Até onde sabemos, este estudo é primeiro estudo a avaliar SLG-VE e SAE juntos em pacientes com acromegalia. O principal achado deste estudo foi a determinação de que as funções sistólicas do VE e do AE em ES eram prejudicadas apesar da preservação das funções sistólicas do VE no ecocardiograma convencional. Outro achado importante foi que o nível de IGF-1, que era um dos parâmetros mais importantes da atividade da doença acromegalia, tinha uma forte correlação com o SAE e o SLG-VE. Além disso, neste estudo, identificou-se que 48% dos pacientes com a acromegalia tinham uma deficiência silenciosa da função sistólica do VE detectada pelo ES, e essa condição foi próxima e independentemente relacionada ao valor de IMVE e nível de IGF-1.

A acromegalia é uma das causas secundárias do HT. A HT e a DM são comuns nesses pacientes devido aos efeitos metabólicos da doença.^7 , 11 , 21 , 22^ Em pacientes com acromegalia, fibrose e alterações hipertróficas ocorrem em graus diferentes miocárdio de AE e VE, sendo que ambos aumentam a frequência de HT e DM, e o nível de IGF-1. A HVE é comum e pode ser vista em 25-85% dos pacientes com acromegalia.^5 , 23 , 24^ Neste estudo, 50% dos pacientes com acromegalia tinham HVE. Em pacientes com acromegalia, as alterações cardíacas devidas ao aumento de hormônios e HT e DM associadas são chamadas miocardiopatia acromegálica (CMP).^25^ A doença consiste em 3 fases: i) aumento da contratilidade do VE e do ventrículo direito (VD) e HVE acompanhado do aumento da IGF-1, ii) disfunção diastólica resultante de redução da elasticidade do VE, iii) aparência típica de cardiomiopatia com dilatação do VE e diminuição de FEVE.^26^ A diminuição de FEVE e dilatação do VE, que são o terceiro estágio da doença, são vistas apenas em 1 a 10% dos pacientes com acromegalia.^11 - 14^ Entretanto, é importante avaliar as funções do VE desses pacientes com um novo método diagnóstico já desde o estágio 1, antes da ocorrência de alterações cardíacas irreversíveis.

A avaliação da função sistólica do VE com SLG-VE é relativamente nova e ainda não é muito comum. O SLG-VE é mais utilizado para avaliar o envolvimento cardíaco subclínico ou silencioso de doenças sistêmicas com FEVE normal.^27 - 30^ A mortalidade cardiovascular aumenta em pacientes com acromegalia.^1 , 2^ Foi demonstrado que os valores de SLG-VE reduzidos estão associados a morte súbita cardíaca e arritmia potencialmente fatal.^31^ O fato de que o ES é facilmente acessível e barato dá uma vantagem importante em relação aos outros métodos. Entretanto, a limitação mais importante é que a qualidade da imagem deve ser muito boa. Embora o ES seja usado frequentemente para a avaliação inicial das funções do VE; nos últimos anos, muitos estudos avaliaram as funções do AE com ecocardiograma strain.^4^

Vários estudos avaliaram o SLG-VE em pacientes com acromegalia e FEVE normal e foram obtidos resultados contraditórios.^5 - 7^ O primeiro estudo foi realizado por Volschan et al.^5^ , em 2017, em 37 pacientes com acromegalia ativa, e detectou-se que o valor de SLG-VE não variou nem aumentou sem significância estatística em comparação aos pacientes de controle. Em outro estudo realizado em 2018, relatou-se que houve uma diminuição no valor do SLG-VE nos pacientes com acromegalia e essa situação estava relacionada a HVE.^7^ Em outro estudo, realizado e publicado muito recentemente pelos mesmos autores, relatou-se que o SLG-VE era reduzido em pacientes com acromegalia, da mesma forma que o estudo anterior.^6^ Este estudo corrobora dois estudos que demonstram que o valor de SLG-VE é reduzido em pacientes com acromegalia. Além dos estudos anteriores, também demonstramos uma redução significativa no SLG-AE no mesmo grupo de pacientes. O valor de corte para o SLG-VE reduzido foi aceito como <20%.^15^ Neste estudo, 48% dos pacientes com acromegalia ficavam <20%. Em outras palavras, as funções sistólicas do VE de metade dos pacientes deste estudo com acromegalia estavam comprometidas. Identificou-se que o IMVE e o IGF-1 tinham uma forte associação com o SLG-VE reduzido em pacientes com acromegalia.^5 , 7^ Os níveis de GH e IGF-1 sérico não estavam relacionados em pacientes com SLG-VE reduzido nos mesmos estudos.^5 , 7^ Neste estudo, assim como no estudo anterior, identificou-se que o IMVE previa pacientes com SLG-VE reduzido. Além disso, os níveis de IGF-1 estavam significativamente relacionados ao SLG-VE reduzido. Neste estudo, identificou-se que cada aumento de 1 gr/m^2^ de IMVE aumentava o risco de SLG-VE reduzido em 6%. Além disso, cada aumento de 1 ng/dL nos níveis de IGF-1 aumenta a probabilidade de diminuição de SLG-VE em 5,6%. O IMVE, que é o achado mais objetivo do envolvimento cardíaco em pacientes com acromegalia, também é o parâmetro mais proximamente associado ao SLG-VE reduzido. Portanto, a intervenção de HT, DM e IMVE o mais cedo possível pode ser a forma mais lógica de retardar a disfunção sistólica futura em pacientes com acromegalia.

O SLG-VE comprometido em pacientes com acromegalia pode ser explicado por dois mecanismos fisiopatológicos. O primeiro é o efeito de HT e DM. A redução do SLG-VE foi demonstrada anteriormente com os efeitos cardíacos de HT e DM mesmo no período assintomático antes de qualquer doença CV.^27 , 28^ Determinou-se que a frequência de HT e DM em pacientes com acromegalia era de 28% e 32%, respectivamente. A prevalência de HT foi significativamente mais alta em pacientes com SLG-VE reduzido. Isso indica que o SLG-VE é afetado pela presença de HT. O segundo mecanismo pode ser a heterotrofia do VE e fibrose miocárdica devido ao aumento do IGF-1 em pacientes com acromegalia sem HT e DM.^23 , 25^ O aumento do valor de IGF-1 pode estar associado à atividade da doença e envolvimento cardíaco. Neste estudo, o nível de IGF-1, que era um dos parâmetros mais importantes da atividade da doença acromegalia, tinha uma forte correlação com o SLG-VE.

Há dados limitados sobre a função e o tamanho do AE em pacientes com acromegalia.^8 , 32^ Em um estudo anterior, relatou-se que o volume e as funções mecânicas do AE eram semelhantes aos dos pacientes de controle saudáveis e os níveis de GH e IGF-1 séricos não foram associados à função mecânica do AE em pacientes com acromegalia.^32^ Em outro estudo recente, um aumento no volume do AE foi relatado em pacientes com acromegalia.^8^ Em nosso estudo, o volume do AE ainda não foi avaliado, mas o diâmetro diastólico do AE era aumentado em pacientes com acromegalia.

Embora não haja estudos na literatura avaliando o SAE com 2D-STE em pacientes com acromegalia, as imagens da deformação de AE foram realizadas em apenas um estudo com 3D-STE.^8^ Kormanyos et al.^8^ relataram uma redução nos valores de deformação segmentar médios e globais, e uma redução no valor de deformação circunferencial de AE. Um achado semelhante foi demonstrado para o átrio direito em outro estudo pelos mesmos autores.^33^ Relatou-se que o nível de IGF-1 e o valor de deformação circunferencial do AE tinham uma correlação positiva.^8^ Nosso estudo foi o primeiro a demonstrar a redução do SAE e sua correlação forte e positiva com o IGF-1, que é um dos parâmetros de atividade da doença.

### Limitações

Como um estudo não randomizado de centro único, esta coorte de pacientes pode ser diferente da de outros centros. O tamanho da amostra é relativamente pequeno e os resultados do estudo precisam ser confirmados ensaios prospectivos multicêntricos grandes no futuro. A mortalidade e a morbidade cardiovasculares são altas em pacientes com acromegalia.^1^ Entretanto, os prognósticos não foram avaliados. Além disso, o efeito do tratamento de SLG-VE e SAE não foi avaliado devido à ausência de um acompanhamento. Neste estudo, não tivemos informações sobre o insight fisiopatológico do SLG-VE reduzido devido à ausência de avaliação histopatológica com biópsia miocárdica. A apneia do sono é comum em pacientes com acromegalia,^1^ e tem um efeito adverso nas funções de VE. Entretanto, não foi possível fazer a polissonografia em todos os pacientes. Os tipos de envolvimento cardíaco mais importantes são HVE e fibrose miocárdica em pacientes com acromegalia. A fibrose miocárdica é mais bem avaliada por imagens por ressonância magnética cardíaca. Se tivesse sido possível realizar as imagens por ressonância magnética cardíaca, teria sido possível avaliar a associação entre realce tardio pelo gadolínio e SLG-VE e SLG-AE. A disfunção diastólica também era comum (50,5%) em pacientes com acromegalia.^34^ Portanto, não avaliamos a disfunção diastólica nesses grupos de pacientes.

Estudos anteriores demonstraram que o volume do VE e do AE e o índice de volume aumentou em pacientes com acromegalia.^6 , 8^ Neste estudo, foram medidos apenas o diâmetro do VE e o diâmetro diastólico final do AE. Se o índice de volume do AE e do VE também tivesse sido medido, poderia haver alterações, especialmente nos parâmetros relacionados a SLG-AE.

## Conclusão

Embora a FEVE seja normal em pacientes com acromegalia, SAE e SLG-VE detectados com 2D-STE são significativamente reduzidos e têm uma relação próxima aos níveis de IGF-1 plasmático. Além do aumento em IMVE, outro achado do envolvimento cardíaco pode ser a redução de SAE e SLG-VE. Portanto, além do ecocardiograma de rotina, SAE e SLG-VE podem ser úteis para avaliar os sinais iniciais de envolvimento cardíaco antes da ocorrência de alterações cardíacas irreversíveis em pacientes com acromegalia.
