# Supplementary material for: Disfunção Ventricular e do Átrio Esquerdo Subclínica em Pacientes com Acromegalia: Um Estudo de Ecocardiograma de Rastreamento de Manchas
Source: Arq Bras Cardiol. 2022 Jan 11;118(3):634–45. [Article in Portuguese] doi: 10.36660/abc.20201174 (PMC8959028; doi:10.36660/abc.20201174)
Supplement: Translation

# Translation: Subclinical Left Atrial and Ventricular Dysfunction in Acromegaly Patients: A Speckle Tracking Echocardiography Study

**Authors:** Hasan Koca, Mevlüt Koc, Hilmi Erdem Sumbul, Yahya Kemal Icen, Erdinc Gulumsek, Fadime Koca, Huseyin Ali Ozturk, Ahmet Oytun Baykan, Onur Kaypakli

**Affiliations:** 1 Turquia Adana Health Practice and Research Center - Department of Cardiology, Adana – Turquia; 2 Turquia Adana Numune Training and Research Hospital, Adana – Turquia; 3 Turquia Adana Health Practice and Research Center - Department of Internal Medicine, Adana – Turquia; 4 Turquia University of Health Sciences - Adana Health Practice and Research Center, Adana – Turquia; 5 Turquia Ortadogu Private Health Hospital - Department of Cardiology, Adana – Turquia; 6 Turquia Mustafa Kemal University Tayfur Ata Sokmen Faculty of Medicine - Department of Cardiology, Hatay – Turquia

**Keywords:** Echocardiography/methods, Acromegaly, Cardiovascular Diseases, Myocardial, Deformability, Diagnostic,Imaging, Stroke Volume

## Abstract

**Background:**

Although it is known that the left ventricular (LV) ejection fraction (EF) measured by echocardiography is preserved in patients with acromegaly, there is not enough information about the LV and left atrial strain (LV-GLS and LAS).

**Objective:**

This study aimed to evaluate the left ventricular (LV) and left atrial (LA) functions with strain echocardiography (SE) in patients with acromegaly.

**Methods:**

This study included 50 acromegaly patients with active disease and 50 healthy controls with similar age, gender, and body surface area. In addition to routine echocardiography examinations, LV-GLS and LAS measurements were performed with SE.

**Results:**

LAS and LV-GLS values were significantly lower in patients with acromegaly (p<0.05 for all). In bivariate analysis, systolic blood pressure, N-terminal prohormone of brain natriuretic peptide, Insulin-like growth factor-1, LA diastolic diameter, and LVMI levels were found to be positively correlated with both LAS and LV-GLS (p <0.05). IGF-1 level was strongly correlated with LAS and LV-GLS (p<0.001 and β=0.5 vs. p<0.001 and β=0.626, respectively); 48% of patients with acromegaly have reduced LV-GLS (<20%). Left ventricular mass-index (LVMI) independently determines the presence of reduced LV-GLS and each 1g/m^2^increase in LVMI level increases the likelihood of reduced LV-GLS by 6%.

**Conclusion:**

Although LV ejection fraction is normal in patients with acromegaly, LAS and LV-GLS values were significantly reduced. Apart from LVMI increase, another finding of cardiac involvement may be LAS and LV-GLS decrease. Therefore, in addition to routine echocardiography, LAS and LV-GLS may be useful to evaluate early signs of cardiac involvement before the occurrence of irreversible cardiac changes.

## Introduction

Acromegaly is a chronic disease characterized by increased Insulin-like growth factor 1 (IGF-1) synthesis in the liver due to a growth hormone (GH) secreting pituitary adenoma, and excessive protein synthesis and excess tissue growth due to these hormones.^1^ Chronic high IGF-1 levels cause specific structural and functional changes.^1^ If left untreated, it causes mortality, and the most common cause of mortality is cardiovascular (CV) diseases.^1 , 2^

Strain is a measure of deformation against a power in a matter. It is defined in radial, circumferential, and longitudinal dimensions. Assessment of myocardial deformation by strain echocardiography (SE), in terms of two-dimensional speckle tracking echocardiography (2D-STE) or tissue Doppler imaging (conventional strain), can provide peerless information on both regional and global ventricular functions.^3^ Deformation imaging can even detect minimal functional changes and provides early stage diagnosis. Left atrium (LA) deformation parameters and left ventricular global longitudinal strain (LV-GLS) have proven to be strongly correlated with LA and LV systolic functions in different clinical scenarios, respectively.^4 - 8^ It has been demonstrated that many patients with normal LV ejection fraction (LVEF) have reduced LV systolic function with the use of LV-GLS.^3 , 9 , 10^

In patients with acromegaly, systolic function is evaluated with LVEF. However, impairment in LVEF is only seen in the later stages of the disease and in the minority of patients.^11 - 14^ Recently, a limited number of studies have evaluated LV-GLS and LA strain in patients with acromegaly and preserved LVEF.^5 - 8^ Contradictory results were obtained in these studies. LV-GLS was found to be decreased in two studies conducted by the same authors;^8 , 9^ whereas, another study reported LV-GLS to be similar to healthy controls.^5^ Evaluation of LA deformation measures in patients with acromegaly with 3D-STE have been performed in a study; however, no clear information was provided regarding the change in global LA strain.^8^

Due to the feasibility of simultaneous measurements of LA deformation and LV-GLS, in addition to traditional echocardiography, this study aimed to evaluate the LV and LA functions with SE in patients with active acromegaly and preserved LVEF.

## Methods

### Study population

In this cross-sectional study, 50 patients (33 male, 17 female; mean age 46.1 ± 6.2 years) with active acromegaly (1-De novo patients, 2-Patients after surgery without remission, 3-Patients on medical treatment without remission) and age, gender, body mass index (BMI), and body surface area (BSA) matched with 50 healthy controls (31 male, 19 female; mean age: 44.6 ± 5.1 years). Patients aged over 18 years with active acromegaly were enrolled in the study. The patients included in the study are shown in the flow-chart ( Figure 1 ). Current guideline information was used for the diagnosis, treatment, and classification of patients with acromegaly.^1^ Remission of acromegaly was defined as a normal glucose-suppressed serum GH less than 0.38 μg/liter (<1 mU/liter), a serum GH less than 1.9 μg/liter (<5 mU/liter), and a normal IGF-1 for age.^1^ Patients with a history of coronary artery disease (CAD) and myocardial infarction, cardiac arrhythmia, systolic heart failure or LVEF <50%, heart valve disease, pulmonary embolism, thyroid dysfunction, pregnancy (known or suspected), active malignancy, and kidney and liver dysfunction, as well as patients who refused to participate in the study were not included in the study. The local ethics committee approved the study protocol (Cukurova University Faculty of Medicine Ethics Committee, 03.05.2019-88), and written informed consent was taken from each participant.

**Figure 1:** – Flow-chart for inclusion and evaluation of patients in the study.

After the assessment of detailed medical history and a complete physical examination, the baseline characteristics of the patients, including age, sex, hypertension (HT), diabetes mellitus (DM), hyperlipidemia, current smoking status, family history of cardiac disease, and medications were recorded for all patients. Participants’ BMI and BSA parameters were calculated.

Glucose, blood urea nitrogen, creatinine, total cholesterol, low density lipoprotein cholesterol, high density lipoprotein cholesterol, triglyceride, aspartate aminotransferase, alanine aminotransferase, white blood cells, hemoglobin, high sensitive C reactive protein, and N-terminal pro-brain natriuretic peptide (NT-proBNP) levels were measured using an automated chemistry analyzer (Abbott Aeroset, MN, USA) with appropriate commercial kits (Abbott). Serum GH was assessed by an automated chemistry analyzer (Abbott Aeroset, MN, USA), using appropriate commercial kits (Abbott) and reference value of GH was between 0.014 -5.219 ng/ml. The serum total IGF-1 level was assessed by an automated chemistry analyzer (Abbott Aeroset, MN, USA), using appropriate commercial kits (Abbott), and the reference value of IGF-1 varies according to age and sex. Serum GH and IGF-1 levels were measured at the same time of echocardiography examination for each subject.

### Echocardiographic assessment

Echocardiographic evaluation was made by using a 2.5-3.5 MHz transducer EPIQ 7C (Philips Healthcare 3000 Minuteman Road, Andover, MA USA). Echocardiographic evaluation was performed within the first week for patients who met the inclusion criteria. Echocardiographic evaluations of all patients were performed in the left lateral decubitus position with electrocardiography and blood pressure monitoring. All images were taken with at least 3 repetitive cycles from the standard parasternal long and short axis, apical 4 chamber, 5 chamber, and 2 chamber views according to the suggestions of the American Society of Echocardiography.^15^ LV diastolic diameter, LV systolic diameter, interventricular septum (IVS) thickness, posterior wall (PW) thickness, LA diastolic diameter were measured from the parasternal long axis image from two-dimensional image windows. Devereux formula was used for LV mass measurement.^16^ Afterwards LV mass-index (LVMI) was calculated by dividing LV mass to BSA. LVMI value > 115 gr/m^2^in men and > 95 gr/m^2^in women were considered LV hypertrophy.^17^

In the SE procedure, all patients were in normal sinus rhythm. LA and LV myocardial deformation parameters were calculated by using STE over two-dimensional gray scale images. Two-dimensional gray scale apical 4 chambers (A4C), apical 2 chambers (A2C), and apical 3 chambers (A3C) were recorded after expiration by holding one’s breath. At least three cardiac cycles were recorded for each image, and attention was paid to consider at least 60-80 fps, according to the guidelines of European Society of Cardiology.^18^ Segments with insufficient image quality and cardiac cycles containing premature beats were excluded from the measurements.

QLAB version 10.5 (Philips, Andover, MA, USA) software was used for LV and LA analyses. The software has automatically followed the wall movements throughout the entire cardiac cycle after the LV endocardium has been marked frame by frame with manual drawing method (manual tracking) on the two-dimensional images. LV-GLS values were calculated from the images with 2D-STE. After manually marking 2 basal and 1 apical parts of LV, the remaining endocardial borders were automatically marked by the software and the appropriate epicardial border was also automatically drawn. When the automatically drawn LV boundaries are not suiTable for analysis, the borders were manually corrected for proper analysis. After analysis, the software divided LV A2C, A3C, A4C recordings to six segments, and an 18-segment model was used to calculate LV-GLS ( Figure 2 ).

**Figure 2:** – Left ventricular global longitudinal strain (LV-GLS) measurement by strain echocardiography in patient with acromegaly.

LA myocardial deformation parameters were also calculated from the images of 2D-STE using LV strain software.^19^ Apical four chamber view images of the LA were obtained using standard anatomic landmarks to ensure optimal acquisition and avoid foreshortening with conventional 2-dimensional echocardiography, at relatively high frame rates (60–80 fps). The LA endocardial border tracing was started at the endocardial border of the mitral annulus, to the LA endocardial border, extrapolating across the pulmonary veins, and/or LA appendage orifices, up to the opposite mitral annulus side by experienced echocardiographers blinded to clinical information. The software then automatically generated an epicardial LA silhouette, which delineated a region of interest in each apical view. Manual adjustment of the region of interest was allowed to encompass the entire LA myocardial layer, followed by automated segmental tracking. After tracking, the LA deformation indices, such as longitudinal strain and its first derivation SR curves, were obtained from a non-foreshortened apical four chamber view.^20^ We used the R wave as a starting point (R-R gating) for deformation analysis. Longitudinal strain and strain rate curves were generated in all segments, and the average of the segments was calculated for the corresponding time points ( Figure 3 ). Using these curves, peak positive LA strain (LAS) and peak systolic strain rates (LASR) were calculated. LAS and LASR represent reservoir function of the LA. All the echocardiographic images were stored digitally and reviewed offline, with deformation measures performed by an experienced cardiologist, blinded to the data, using Philips QLAB version 10.5 software analysis.

**Figure 3:** – Left atrial deformation parameter measurement by strain echocardiography in patient with acromegaly.

### Statistical analyses

Statistical analyses were conducted using SPSS, version 23.0, (SPSS Inc. Chicago, Illinois). Data are expressed as mean ± SD for continuous variables and percentage for categorical variables. The Shapiro-Wilk test was used to test normality, and a p-value >0.05 was defined as normally distributed data. Continuous variables that showed normal distribution were compared using the Student’s t test and ANOVA, whereas the Mann-Whitney U and Kruskal-Wallis tests were used for non-normally distributed samples. Categorical variables and frequencies were compared by means of the chi-square test. Statistical significance was defined as a p-value <0.05 for all comparisons. In our study, parameters that differed in patients with <20% for LV-GLS were found in univariate analysis. Therefore, Backward: LR logistic regression analysis was performed to determine the parameters that independently determined patients with <20% for LV-GLS. Pearson’s and Spearman’s correlation were used to examine the relationship between continuous variables. Variables with a p-value <0.05 in the bivariate analysis were tested in the linear regression analysis. Results were expressed as the p-value and hazard ratio (HR) in a 95% CI.

## Results

Fifty-eight patients with active acromegaly were included in the study. Eight patients who met the exclusion criteria and could not perform ideal echocardiographic examination were excluded from the study. Forty-five patients included in the study were De novo acromegaly patients. The other three patients were after surgery without remission and two patients were on medical treatment without remission. The study data were divided into two groups, with and without acromegaly (healthy controls). Cohen kappa values that evaluate interobserver and intraobserver variability were over 90% for all echocardiography measurements.

### Demographic, clinical, and laboratory data

When demographic data were compared between the study groups; age, gender, BMI, and BSA were similar between the groups. The frequency of HT and DM in patients with acromegaly was determined to be 28% and 32%, respectively. In terms of clinical parameters, systolic and diastolic blood pressures and heart rate were higher in patients with acromegaly. Plasma glucose, NT-proBNP, IGF-1, and growth hormone levels were found to be significantly higher in patients with acromegaly. Other laboratory parameters were similar between the two groups ( Table 1 ).

**Table 1:** – Demographic, clinical, and laboratory parameters of acromegaly patients and healthy controls

Variable	Acromegaly patients n=50	Healthy controls n=50	p
Age (year)	46.1 ± 6.2	44.6 ± 5.1	0.295
Gender (female)	17	19	0.418
Hypertension, n (%)	14 (28%)	–	–
Diabetes mellitus, n (%)	16 (32%)	–	–
Current smoker, n (%)	15 (30%)	–	–
Hyperlipidemia, n (%)	7 (14%)	–	–
Systolic blood pressure (mmHg)	130 ± 19	110 ± 10	<0.001
Diastolic blood pressure (mmHg)	81 ± 11	67 ± 6.4	<0.001
Heart rate (pulse/minute)	81 ± 11	67 ± 4.1	<0.001
Body mass index (kg/m^2^)	28.1 ± 2.3	27.6 ± 1.6	0.164
Body surface area (m^2^)	2.01 ± 0.10	2.00 ± 0.09	0.569
White blood cell (µL)	7.3 ± 1.9	7.5 ± 1.6	0.656
Hemoglobin (gr/dL)	13.1 ± 1.8	12.9 ± 1.2	0.420
Plasma glucose (mg/dL)	109 ± 23	92 ± 5.6	<0.001
Blood urea nitrogen (mg/dL)	32.9 ± 16.6	29.5 ± 4.1	0.149
Creatinine (mg/dL)	0.75 ± 0.42	0.64 ± 0.10	0.138
Total cholesterol (mg/dL)	197 ± 59	217 ± 60	0.095
Low density lipoprotein (mg/dL)	135 ± 45	148 ± 44	0.157
High density lipoprotein (mg/dL)	44.3 ± 15.3	48.2 ± 8.1	0.125
Triglyceride (mg/dL)	165 ± 77	191 ± 108	0.180
Aspartate aminotransferase (u/L)	20.6 ± 7.4	18.9 ± 3.4	0.143
NT-proBNP (pg/mL)	365 ± 297	74 ± 6.7	<0.001
hs-CRP (mg/dL)	1.69 ± 1.35	0.43 ± 0.31	<0.001
Alanine aminotransferase (u/L)	16.8 ± 8.9	15.9 ± 2.9	0.298
IGF-1 (ng/dL)	376 ± 181	72 ± 7.5	<0.001
Growth hormone (ng/mL)	9.21 ± 14.4	1.01 ± 0.52	<0.001

*hs-CRP: High sensitive C reactive protein; IGF-1: Insulin-like growth factor 1; NT-proBNP: N-terminal pro-brain natriuretic peptide.*

### Echocardiographic data

IVS and PW end-diastolic thickness and LVMI values were found to be significantly higher in patients with acromegaly ( Table 2 ). LV diameters and LVEF values were found to be similar between the groups with and without acromegaly. LAS and LV-GLS values were significantly lower in patients with acromegaly ( Figure 4 - 5 ).

**Table 2:** – Echocardiography parameters of acromegaly patients and healthy controls

Variable	Acromegaly patients n=50	Healthy controls n=50	p
IVS end-diastolic thickness (mm)	12.3 ± 1.92	9.9 ± 1.21	<0.001
PW end-diastolic thickness (mm)	11.9 ± 1.32	9.7 ± 1.01	<0.001
LV end-diastolic dimension (mm)	46.7 ± 4.5	47.3 ± 4.3	0.516
LV end-systolic dimension (mm)	31.1 ± 4.2	31.5 ± 4.2	0.656
LA end-diastolic dimension (mm)	35.3 ± 4.2	33.1 ± 2.6	0.002
LV ejection fraction (%)	57.8 ± 4.1	58.9 ± 5.3	0.259
LV mass index (gr/m2)	108 ± 28	82± 17	<0.001
LV hypertrophy, n (%)	25 (50%)	0 (0%)	<0.001
LAS (%)	21.5 ± 1.36	23.5 ± 1.06	<0.001
LV-GLS (%)	– 20.4 ± 1.45	– 22.8 ± 0.83	<0.001
LV-GLS < 20%, n (%)	24 (48%)	0 (0%)	<0.001

*IVS: Interventricular septum; LA: Left atrial; LV-GLS: Left ventricular global longitudinal strain; LV: Left ventricular; PW: posterior wall; LAS: peak positive LA strain.*

**Figure 4:** – The Boxplot graphic showed that left ventricular global longitudinal strain (LV-GLS) in patient with acromegaly and healthy controls.

**Figure 5:** – The Boxplot graphic showed that peak positive LA strain (LAS) in patient with acromegaly and healthy controls.

When the limit value was taken as <20% for LV-GLS, it was found that LV-GLS levels were decreased in 48% of the acromegaly group. When the demographic, clinical, and laboratory data of patients with decreased and normal LV-GLS acromegaly were compared, it was determined that patients with low LV-GLS had higher rates of HT (45.8% vs 11.5% and p = 0.008) and higher LVMI values (123 gr/m^2^vs 94.6 gr/m^2^and p <0.008). In the multivariate logistic regression analysis, it was found that the LVMI value and IGF-1 levels independently predict the reduced LV-GLS value (p = 0.003, OR: 1.060 and CI: 1.019 - 1.102 and p = 0.012, OR: 1.056 and CI: 1.023 - 1.098). According to this analysis, every 1 gr/m^2^increase in LVMI value and every 1 ng/dL increase in IGF-1 level increases the probability of decreased LV-GLS by 6% and 5.6%, respectively.

### Determination of LA deformation measurement

Correlation analysis was performed to determine the parameters associated with LA deformation. Parameters related to LAS in correlation analysis were summarized in Table 3 . Linear regression analysis was performed to determine the presence of independent relationships of LAS. In linear regression analysis; systolic BP, NT-proBNP, IGF-1, LA end-diastolic diameter, and LVMI were found to be positively and significantly associated with LAS. Statistically, the strongest correlation was found to be between LAS and IGF-1 levels ( Table 3 and Figure 6 ).

**Table 3:** – The parameters associated with LA-GLS and linear regression analysis for parameters significantly correlated with LAS

	Univariate analysis		Multivariate analysis	
	p	r	p	β
Systolic blood pressure	<0.001	0.427	0.001	0.278
Diastolic blood pressure	<0.001	0.362	0.470	0.102
Heart rate	<0.001	0.360	0.840	0.023
Plasma glucose	<0.001	0.418	0.255	0.133
Creatinine	0.018	0.225	0.712	0.064
NT-proBNP	<0.001	0.445	0.013	0.237
IGF-1 (ng/dL)	<0.001	0.531	<0.001	0.531
Growth hormone (ng/mL)	0.025	0.225	0.408	0.096
LA end-diastolic dimension	<0.001	0.662	<0.001	0.378
LV mass index	<0.001	0.623	<0.001	0.503

*LA: Left atrial; LAS: peak positive LA strain; LV: left ventricular; IGF-1: Insulin-like growth factor 1; NT-proBNP: N-terminal pro-brain natriuretic peptide. R^2^_adjusted_=0,684 and p< 0.001 in multivariate analyses.*

**Figure 6:** – Scatter plot diagram of the relationship of peak positive LA strain (LAS) with insulin-like growth factor 1 (IGF-1). A 10-logarithmic scale of IGF-1 value was obtained.

### Determination of LV-GLS-related parameters

Correlation analysis was performed to determine the parameters associated with LV-GLS. Parameters related to LV-GLS in the correlation analysis were summarized in Table 4 . Linear regression analysis was performed to determine the presence of independent relationships of LV-GLS related parameters. In linear regression analysis; systolic blood pressure, NT-proBNP, IGF-1, LA end-diastolic diameter and LVMI were found to be positively and significantly associated with the LV-GLS value. Statistically, the strongest correlation was found to be between the LV-GLS and IGF-1 levels ( Table 4 and Figure 7 ).

**Table 4:** – The parameters associated with LV-GLS and linear regression analysis for parameters significantly correlated with LV-GLS

	Univariate analysis		Multivariate analysis	
	p	r	p	β
Age	0.026	0.223	0.844	0.017
Body mass index	0.033	0.213	0.256	0.092
Systolic blood pressure	<0.001	0.509	<0.001	0.300
Diastolic blood pressure	<0.001	0.462	0.605	0.076
Heart rate	<0.001	0.408	0.426	0.081
Plasma glucose	<0.001	0.442	0.172	0.146
Creatinine	0.015	0.243	0.263	0.090
NT-proBNP	<0.001	0.478	0.011	0.176
IGF-1	<0.001	0.626	<0.001	0.626
Growth hormone (ng/mL)	<0.001	0.429	0.050	0.207
LA end-diastolic dimension	0.001	0.341	0.009	0.199
LV mass index	<0.001	0.623	<0.001	0.548

*IGF-1: Insulin-like growth factor 1; NT-proBNP: N-terminal pro-brain natriuretic peptide; LV-GLS: Left ventricular global longitudinal strain. R^2^_adjusted_=0,641 and p< 0.001 in multivariate analyses.*

**Figure 7:** Scatter plot diagram of the relationship of left ventricular global longitudinal strain (LV-GLS) with Insulin-like growth factor 1 (IGF-1). A 10-logarithmic scale of IGF-1 value was obtained.

## Discussion

To the best of our knowledge, our study is the first study to evaluate LV-GLS and LAS together in patients with acromegaly. The main finding of our study was that LV and LA systolic functions in SE were found to be impaired despite the preservation of LV systolic functions in conventional echocardiography. Another important finding was that the IGF-1 level, which is one of the most important parameters of acromegaly disease activity, was strongly correlated with LAS and LV-GLS. In addition, in our study, it was found that 48% of patients with acromegaly had silent impaired LV systolic function detected with SE and this condition was closely and independently related to LVMI value and IGF-1 level.

Acromegaly is one of the secondary causes of HT. HT and DM are common in these patients due to the metabolic effects of the disease.^7 , 11 , 21 , 22^ In patients with acromegaly, fibrosis and hypertrophic changes occur in different degrees of LV and LA myocardium, with both increasing frequency of HT and DM, and an increasing IGF-1 level. LVH is common and can be seen in 25-85% of all patients with acromegaly.^5 , 23 , 24^ In our study, 50% of the patients with acromegaly had LVH. In patients with acromegaly, cardiac changes due to increased hormones and associated HT and DM are called acromegalic cardiomyopathy (CMP).^25^ This disease consists of 3 phases; i) an increase in LV and right ventricle (RV) contractility and LVH accompanied by increased IGF-1, ii) diastolic dysfunction as a result of decreased LV elasticity, iii) typical cardiomyopathy appearance with LV dilatation and decreased LVEF.^26^ LVEF decrease and LV dilatation, which is the 3rd stage of the disease, are seen in only 1-10% of the patients with acromegaly.^11 - 14^ However, it is important to evaluate LV functions of these patients with a new diagnostic method as early as stage 1, before irreversible cardiac changes occur.

Evaluation of LV systolic function with LV-GLS is relatively new and still quite uncommon. LV-GLS is mostly used to evaluate subclinical or silent cardiac involvement of systemic diseases with normal LVEF.^27 - 30^ Cardiovascular mortality increases in patients with acromegaly.^1 , 2^ Decreased LV-GLS values have proven to be associated with sudden cardiac death and life-threatening arrhythmia.^31^ The fact that SE is easily accessible and inexpensive gives it an important advantage over other methods. However, the most important limitation is that the image quality must be very good. Although SE was used frequently to evaluate LV functions in the first place; in the last few years, many studies evaluated LA functions with strain echocardiography.^4^

Several studies have evaluated LV-GLS in patients with acromegaly and normal LVEF, and contradictory results have been obtained.^5 - 7^ The first study was conducted by Volschan et al.^5^ in 2017 in 37 patients with active acromegaly, and it was found that the LV-GLS value did not change or even increased with no statistical significance when compared to healthy controls. In another study conducted in 2018, it was reported that there was a decrease in LV-GLS value in acromegaly patients, and this situation was related to LVH.^7^ In another study conducted and published very recently by the same authors, LV-GLS was reported to be lower in patients with acromegaly, similar to the previous study.^6^ Our study supports two studies showing that the LV-GLS value is decreased in patients with acromegaly. In addition to previous studies, we also showed a significant decrease in LA-GLS in the same patient group. The cut-off value for reduced LV-GLS was accepted as <20%.^15^ In our study, 48% of the patients with acromegaly were under <20%. In other words, LV systolic functions of half of the patients with acromegaly were impaired. Increased LVMI and IGF-1 were found to be strongly associated with decreased LV-GLS in acromegaly patients.^5 , 7^ Serum IGF-1 and GH levels were not related in patients with reduced LV-GLS in the same studies.^5 , 7^ In our study, similar to the previous study, LVMI was found to predict patients with reduced LV-GLS; moreover, IGF-1 levels were significantly related to reduced LV-GLS. In our study, it was found that every 1 gr/m^2^increase in LVMI increased the risk of reduced LV-GLS by 6%. In additional to this, every 1 ng/dL increase in IGF-1 level increased the probability of decreased LV-GLS by 5.6%. LVMI, which is the most objective finding of the cardiac involvement in patients with acromegaly, is also the most closely associated parameter with decreased LV-GLS. Therefore, intervention to HT, DM, and LVMI as early as possible may be the most logical way to delay future systolic dysfunction in patients with acromegaly.

Impaired LV-GLS in patients with acromegaly can be explained by two pathophysiological mechanisms. The first is the effect of HT and DM. LV-GLS reduction has been demonstrated previously with the cardiac effects of HT and DM even in the asymptomatic period before any CV disease.^27 , 28^ In our study, the prevalence of HT and DM in patients with acromegaly is 28% and 32%, respectively. The prevalence of HT was significantly higher in patients with decreased LV-GLS. This indicates that LV-GLS is affected by the presence of HT. The second mechanism may be LV heterotrophy and myocardial fibrosis due to increased IGF-1 in patients with acromegaly without HT and DM.^23 , 25^ An increased IGF-1 value may be associated with disease activity and cardiac involvement. In our study, the IGF-1 level, which is one of the most important parameters of acromegaly activity, was strongly correlated with LV-GLS.

There is limited data on LA function and size in patients with acromegaly.^8 , 32^ In a previous study, it was reported that LA volume and mechanical functions were similar to healthy controls, and serum IGF-1 and GH levels were not associated with LA mechanical function in patients with acromegaly.^32^ In another recent study, an increase in LA volume was reported in patients with acromegaly.^8^ In our study, LA volume was not evaluated, but LA diastolic diameter was increased in patients with acromegaly.

Although there is no study in the literature evaluating LAS with 2D-STE in acromegaly patients, LA strain imaging was performed in only one study with 3D-STE.^8^ Kormanyos et al.^8^ reported that LA global and mean segmental strain values were increased and LA circumferential strain values was decreased. A similar finding was demonstrated for right atrium in another study by the same authors.^33^ It has been reported that the IGF-1 level and the LA circumferential strain value were positively correlated.^8^ Our study was the first to demonstrate a decrease in LAS, and its strong and positive correlation with IGF-1, which is one of the disease activity parameters.

### Limitations

As a single-center non-randomized study, our patient cohort might be different from other centers. The sample size is relatively small and our results need to be confirmed in future, large multi-center prospective trials. CV mortality and morbidity are high in patients with acromegaly.^1^ However; we did not evaluate the prognosis. In addition, the effect of treatment on LV-GLS and LAS was not evaluated due to the absence of follow-up. In our study, we did not have the information regarding the pathophysiologic insight of reduced LV-GLS due to the lack of histopathological evaluation with myocardial biopsy. Sleep apnea is common in patients with acromegaly,^1^ and it has an adverse effect on LV functions. However, we were not able to do polysomnography in all patients. The most important types of cardiac involvement are LVH and myocardial fibrosis in patients with acromegaly. Myocardial fibrosis can be best assessed with cardiac magnetic resonance imaging. If we had been able to perform cardiac magnetic resonance imaging, we would have been able to evaluate the association of late gadolinium enhancement with LA-GLS and LV-GLS. Diastolic dysfunction was also common (50.5%) in patients with acromegaly.^34^ Therefore; we did not evaluate the diastolic dysfunction in these patients groups.

It was shown in previous studies that LA and LV volume and volume index increased in patients with acromegaly.^6 , 8^ In our study, we only measured LV diameter and LA end-diastolic diameter. If we also measured the LA and LV volume index, there could be changes especially in the parameters related to LA-GLS.

## Conclusion

Although LVEF is normal in patients with acromegaly, LAS and LV-GLS detected with 2D-STE are significantly lower and are closely related to plasma IGF-1 levels. Apart from an increase in LVMI, another finding of cardiac involvement may be the decrease in LAS and LV-GLS. Therefore, in addition to routine echocardiography, LAS and LV-GLS may be useful to evaluate early signs of cardiac involvement before the occurrence of irreversible cardiac changes in patients with acromegaly.
